# Linking Diet to Colorectal Cancer: The Emerging Role of MicroRNA in the Communication between Plant and Animal Kingdoms

**DOI:** 10.3389/fmicb.2017.00597

**Published:** 2017-04-05

**Authors:** Manuela Del Cornò, Gloria Donninelli, Lucia Conti, Sandra Gessani

**Affiliations:** ^1^Department of Hematology, Oncology and Molecular Medicine, Istituto Superiore di SanitáRome, Italy; ^2^Center for Gender-Specific Medicine, Istituto Superiore di SanitáRome, Italy

**Keywords:** microRNA, colorectal cancer, inter kingdom communication, diet, bioactive food components, epigenetic mechanisms

## Abstract

Environmental and lifestyle factors, including diet and nutritional habits have been strongly linked to colorectal cancer (CRC). Of note, unhealthy dietary habits leading to adiposity represent a main risk factor for CRC and are associated with a chronic low-grade inflammatory status. Inflammation is a hallmark of almost every type of cancer and can be modulated by several food compounds exhibiting either protective or promoting effects. However, in spite of an extensive research, the underlying mechanisms by which dietary patterns or bioactive food components may influence tumor onset and outcome have not been fully clarified yet. Growing evidence indicates that diet, combining beneficial substances and potentially harmful ingredients, has an impact on the expression of key regulators of gene expression such as the non-coding RNA (ncRNA). Since the expression of these molecules is deranged in chronic inflammation and cancer, modulating their expression may strongly influence the cancer phenotype and outcomes. In addition, the recently acquired knowledge on the existence of intricate inter-kingdom communication networks, is opening new avenues for a deeper understanding of the intimate relationships linking diet to CRC. In this novel scenario, diet-modulated ncRNA may represent key actors in the interaction between plant and animal kingdoms, capable of influencing disease onset and outcome. In this review, we will summarize the studies demonstrating a link between bioactive food components, including food-derived, microbiota-processed, secondary metabolites, and host ncRNA. We will focus on microRNA, highlighting how this plant/animal inter-kingdom cross-talk may have an impact on CRC establishment and progression.

## Introduction

Colorectal cancer (CRC) is the second most commonly diagnosed cancer in women and the third in men worldwide (Ferlay et al., 2015) that presents one of the highest rates of morbidity and mortality worldwide (Siegel et al., 2016). The number of new cases of cancer including CRC, has been increasing in the last decades (Torre et al., 2016), and such increased incidence has been attributed to environmental factors, i.e., adoption of Western diets and lifestyles (Haggar and Boushey, 2009). Chronic intestinal inflammation (Ekbom et al., 1990; Feagins et al., 2009) and obesity (Pietrzyk et al., 2015) represent additional risk factors associated with increased CRC incidence. Obesity has become a major threat to public health because of its high global prevalence and association with an increased risk of developing chronic diseases (Abdoullaye et al., 2010). Obesity affects over half a billion adults worldwide, with approximately 3.5 million attributable deaths each year (WHO, 2015). Similarly to gender, race, dietary habits or smoking history, obesity not only represents a risk factor for several tumors including CRC (Park et al., 2014; Parkin et al., 2014), but contributes to 3–20% of cancer deaths in Western populations (Renehan et al., 2008; Beddy et al., 2010). Abdominal rather than total adiposity is associated with a 1.5− to 3.5−fold increased risk of developing CRC as compared to lean individuals (Bardou et al., 2013).

Notably, dietary components influence inflammatory processes, and many types of cancer, including CRC, can be prevented/delayed through healthy life styles (Turati et al., 2012). The most extensive review of the existing evidence connecting diet and cancer is the 2007 World Cancer Research Fund/American Institute for Cancer Research (WCRF/AICR; Lozcano-Ponce, 2009) report and its subsequent update (Wiseman, 2008; Martinez-Gonzalez et al., 2015; Norat et al., 2015).

CRC initiation/progression results from the accumulation over-time of genetic changes in oncogenic/oncosuppressor genes in colonic epithelium, with epigenetic alterations recognized as significant contributors to cancer development. CRC “epigenome” assessment revealed that virtually all CRC have aberrantly methylated genes and altered microRNA (miR) expression (Okugawa et al., 2015). Dysregulation of miR and their mRNA targets contributes to the initiation/progression of colon carcinogenesis as well as to invasion, angiogenesis, and metastasis (Ramalingam et al., 2015; Ress et al., 2015). Interestingly, bioactive food ingredients exert not only direct effects on carcinogenesis but likely influence cancer development indirectly by affecting gut microbiota composition/metabolism, and by epigenetically regulating gene expression. Complex interactions among food components and histone modifications, chromatin remodeling, DNA methylation and non-coding RNA (ncRNA) expression lead to a dynamic regulation of gene expression controlling cellular phenotype (Milagro et al., 2013). Dietary changes also affect gut microbiota in terms of relative abundance of microbial species. In turn, microbiota influences the conversion of food components and fibers into metabolites acting as epigenetic regulators in cancer, as well as nutrient uptake and epithelial resilience (Ha et al., 2014; O’Keefe et al., 2015; Bultman, 2016). Additionally, the presence of cancer-associated circulating miR (Schou et al., 2016) and the growing attention to xeno-miR, absorbed with food ingestion (Fabris and Calin, 2016), further highlight the complexity of inter-kingdom communication and its potential role in the balance between homeostasis and disease.

In this review, we focus on diet-induced modulation of host miR and discuss their potential contribution to CRC. In the following paragraphs we will examine different outcomes of this plant/animal inter-kingdom cross-talk including both preventing and pro-tumorigenic effects.

## The Dual Effect of Dietary Patterns and/or Bioactive Food Components in CRC: The Role of miR

It has long been known that modifications of diet can prevent, slow, and even reverse some disease-associated events, and growing evidence suggests that one of the mechanisms by which nutrients and bioactive compounds affect metabolic traits is epigenetics. Various diets and dietary interventions, including high-fat diets (HFD) and caloric restriction (CR), as well as bioactive nutrients and plant derivatives, have been associated with epigenetic changes that alter cellular signaling (Hardy and Tollefsbol, 2011; Garcia-Segura et al., 2013) and may have an impact on CRC development (Bultman, 2016; Lee et al., 2016). There is currently overwhelming evidence that consumption of red and processed meat as well as animal fat, typical of Western style diets, increases CRC risk (Bernstein et al., 2015). Conversely, bioactive dietary molecules, such as ω3 polyunsaturated fatty acids (PUFA), curcumin, fermentable fibers (basic components of the Mediterranean diet), folate, calcium, vitamin D, and physical activity exert chemoprotective effects (Hou et al., 2016). Although the mechanisms underlying the role of food in preventing or favoring CRC are not fully elucidated, growing evidence indicates that at least some of them involve miR (Gavrilas et al., 2016; Hou et al., 2016).

A summary of the main results achieved in both *in vitro* and *in vivo* models is shown in **Table 1**.

**Table 1:** Protective versus promoting effects of dietary patterns and components in CRC.

TUMOR PROTECTIVE/PREVENTIVE EFFECTS						

**Diet/dietary component**	**Source/in combination with (+)**	**Cell line/animal model**	**microRN↑a**	**microRNA↓**	**Regulated pathway(s)**	**Reference**
**Polyphenols**
Resveratrol		SW480	22 miR (i.e., miR-146b-5p, -1, -663)	26 miR (i.e., miR-21, -196a, -25, -17, -92a)	↓TGFβ signaling, E-cadherin, Dicer ↑ PDCD4, PTEN	Tili et al., 2010
	Botanical extract/+ Quercetin	HT29		miR-27a	↓Sp-1, -3, -4, survivin ↑ZBTB10	Del Follo-Martinez et al., 2013
	+ EGCG, α-mangostin, or 5-FU	SW480, DLD1, COLO201	miR-34a		↓E2F3, SIRT1	Kumazaki et al., 2013
		Mouse (sporadic CRC)	miR-96		↓KRAS	Saud et al., 2014
Curcumin	+5-FU	RKO, HCT116, SW480, 5FUR Mouse xenograft model	miR-200b, -200c, -141, -429, -34, -101	miR-21, -27a, -20a, -17-5p	↓Sp-1, -3, -4, ROS, survivin, Bcl2, EGFR, NF-κB, cyclin D, BMI1, E2H2, SUZ12 ↑ PDCD4, ZBTB4, ZBTB10	Mudduluru et al., 2011; Gandhy et al., 2012, Toden et al., 2015b
	+ Boswellic acid	HCT116, SW480 mouse xenograft model	miR-34a	miR-27a		Toden et al., 2015a
CDF		HCT116, HT29, SW620	miR-34a, -34c	miR-21	↓Akt, Notch-1 ↑PTEN	Roy et al., 2012, 2013, Yu et al., 2013
Flavonoids	Cowpea (*Vigna unguiculata*)	CCD18Co	miR-126		↓VCAM	Ojwang et al., 2015
	Yaupon holly (*Ilex vomitoria*)	HT29, CCD18Co	miR-146a		↓NF-κB	Noratto et al., 2011
Ellagic acid and ellagitannins, urolithins	Pomegranate extract (*Punica granatum*)	HT29 Rat	miR-126		↓VCAM-1, PI3K/Akt, mTOR	Banerjee et al., 2013
	Pomegranate extract (*Punica granatum*)	Human colon biopsies	General induction of miR attributable to the surgery	miR-646, -1249, -135b-5p/3p, -92b-5p, -765, -496, -181c-3p, -18a-3p		Nunez-Sanchez et al., 2015
		Caco2, HT29, CCD18Co	miR-215	miR-224	↑CDKN1A	Gonzalez-Sarrias et al., 2016
α-Mangostin	*Garcinia mangostana*/+5-FU	DLD1	miR-143		↓ERK5, c-Myc	Nakagawa et al., 2007
Red wine polyphenolics	*Vitis aestivalis* hybrid	CCD18Co	miR-126		↓NF-κB, ICAM-1, VCAM-1, PECAM-1	Angel-Morales et al., 2012
Proanthocyanidins	Grape seed extract	Mouse	miR-19a, -20a, -let7a, Snord 68	miR-205, -135b, -196a, -21, -148a, -103	↓NF-κB, COX2, INOS, VEGF ↑Ago2	Derry et al., 2013
Canolol, 4-vinyl-2, 6-dimethoxyphenol	Crude canola oil	Mouse	miR-7		↓COX-2/PGE2	Cao et al., 2015
**Fatty acids**
DHA		SGC7901, BGC823, MGC803, HCT116, HCT8, Caco2, HepG2	miR-15b, -16, -141-3p, -221-3p, -192, -30c, 1283, -let7f, -181a, -1	miR-21, -30a	↓Bcl2 ↑ TNF-α genes related to lipid metabolism and cancer biology	Sun et al., 2013; Gil-Zamorano et al., 2014, Fluckiger et al., 2016
ω3 PUFA versus ω6 PUFA	Fish oil-pectin diet compare to control corn oil-cellulose diet	AOM-induced mice and rats	miR-10a, -21, -26b, -200a/c, -203, -16, -19b, -27b, -93,- let7d, -15b, -107, -191, -324-5p, -218		↓PDE4B, PTK2B, TCF4, IGF1R, BACE1	Davidson et al., 2009; [Bibr B67][Bibr B66]
	Walnut diet	Mouse xenograft model	miR-297a^∗^	miR-1903, -467c, -3068		Tsoukas et al., 2015
Butyrate		HCT116, HCT29 Human colon biopsies	18 miR (i.e., miR-106b)	26 miR (i.e., miR-17-92, -18a/b, -19a/b, -25, -20a)	↑ PTEN, CDKN1A, CDKN1C, BCL2L11 ↓c-Myc	Hu et al., 2011, 2015, Humphreys et al., 2013
**Diets**					
Calorie restriction diet (30%)		AOM-induced mice	miR-150, -351, -16-2a, let7f, -34	miR-155		Olivo-Marston et al., 2014
**TUMOR PROMOTING EFFECTS**					
**Diets**					
High-fat diet		AOM/DSS-induced mice	miR-425, -196a, -155	miR-150, -351, -16-2a, -let7f, -34, -138, -143, -145	↑c-Myc, KRAS (EGFR-mediated)	Dougherty et al., 2009; Zhu et al., 2011, Olivo-Marston et al., 2014
High red meat diet	+ Amylose maize starch	Healthy human volunteers	miR-17, -18a, -19a, -20a, -19b, -92a, -21		↓ CDKN1A	Humphreys et al., 2014
2-amino-1-methyl-6-phenylimidazo [4,5-b] pyridine/HFD	+ Dietary spinach	Rat	miR-126, -145, -21	miR-215, -29c, -98, -let7 family	↑ SOX2, HMGA2, β-Catenin, Cyclin D1, c-Myc, Lin28A/B ↓p53	Parasramka et al., 2012
**Methyl donor nutrients**					
Folate (serum level or exogenously added)		Human subjects, Caco2, HT29, HCT116	miR-21			Beckett et al., 2015

*A summary of the main studies showing the protective versus promoting effects of dietary patterns and bioactive food compounds on miR expression in *in vitro* and *in vivo* CRC models*.

## Protective Effects

Dietary patterns and some bioactive food components including polyphenols, ω3 PUFA, and short chain fatty acids (SCFA) exhibit a chemopreventive role against CRC. Among phytochemicals, polyphenols are ubiquitous secondary metabolites found in fruits and vegetables, whole grain cereals, and beverages (e.g., tea, coffee, and wines). Initial studies showed that resveratrol (RES), a stilbenoid found in dried fruits, berries, peanuts and especially in grapes, modulates the levels of miR targeting both oncogenes and tumor suppressor genes. In particular, RES increases the levels of miR-663, a tumor suppressor miR targeting TGFβ1 transcripts (Tili et al., 2010). Likewise, α-mangostin (α-M), a xanthone from mangosteen pericarps, exhibits anti-proliferative/pro-apoptotic effects by targeting ERK5/c-Myc via miR-143 (Nakagawa et al., 2007). Subsequent studies investigated the effects of phytochemical combinations, including epigallocatechin-3-gallate (EGCG), RES, quercetin, and α-M, or phytochemical association with anti-cancer drug 5-fluorouracil (5-FU). In this regard, it was demonstrated that the combination of substances naturally occurring in the colonic lumen after ingestion of polyphenol-containing food, such as RES and quercetin, has a pro-apoptotic effect on CRC cells (Del Follo-Martinez et al., 2013). The interplay of RES and quercetin with the miR-27a-ZBTB10 axis, repressing Sp-1 activity, was identified as one possible underlying mechanism. Combinations of RES with EGCG or α-M acted as chemo-sensitizer through up-regulation of miR-34a and down-modulation of its target genes E2F3 and Sirt1, leading to apoptosis induction (Kumazaki et al., 2013). Lastly, α-M and 5-FU exerted a synergistic effect on growth inhibition (Nakagawa et al., 2007). Additional *in vitro* evidence showed that the flavonol-rich fractions from botanical extracts, as well as red wine polyphenolics, inhibited the generation of reactive oxygen species (ROS) and NF-κB activation in colon cells by inducing miR-126 and miR-146a (Noratto et al., 2011; Angel-Morales et al., 2012; Ojwang et al., 2015).

The therapeutic potential of pomegranate (PO), of its main polyphenolic compounds (ellagic acid, and ellagitannins) as well as of their gut microbiota-derived metabolites (urolithins), has been reported *in vitro* and *in vivo* CRC models. Gonzalez-Sarrias et al. (2016) found relevant changes in cancer markers and identified the induction of p21waf1/Cip1 (CDKN1A) as a common step underlying urolithin anticancer properties. Interestingly, miR-224 down-regulation or miR-215 up-regulation was associated with CDKN1A induction (Gonzalez-Sarrias et al., 2016).

Among *in vivo* tested compounds, RES and proanthocyanidin-rich extracts prevented tumorigenesis in sporadic CRC models by suppressing Kras activity (Saud et al., 2014) and inflammatory pathways (Derry et al., 2013), through miR modulation. Likewise, PO polyphenols exerted cytotoxic and anti-inflammatory effects in experimentally induced colon carcinogenesis in rat and in CRC cells. Interaction of PO with miR-126/VCAM-1 and miR-126/PI3K/AKT/mTOR axes were identified as mechanisms that at least in part mediate the anti-inflammatory/anti-proliferative activities of these compounds (Banerjee et al., 2013). Interestingly, in a controlled human trial, Nunez-Sanchez et al. (2015) demonstrated that PO consumption affected specific colon miR other than miR-126. Lastly, canolol, an anti-oxidant from canola oil, inhibited gastric tumor by blocking COX-2/PGE2/EP2 signaling pathway in mouse models. Interestingly, COX-2 is a functional target of miR-7, a tumor suppressor miR reactivated after canolol administration (Cao et al., 2015).

Another compound relevant to CRC prevention is curcumin, a bioactive ingredient of turmeric, with anti-inflammatory, antioxidant, and anti-carcinogenic properties (Patel et al., 2010). Similarly to other polyphenols, modulation of miR expression by curcumin in cell lines has been reported as a mechanism underlying the effects of this compound (Reuter et al., 2011). Several studies highlighted miR-21, an onco-miR overexpressed in many tumors, as an important target of curcumin activity. Curcumin inhibited miR-21 expression, tumor growth, invasion and *in vivo* metastasis, and stabilized its tumor suppressor target Pdcd4 in CRC cells (Mudduluru et al., 2011). Likewise, curcumin-difluorinated (CDF), a curcumin analog with a greater bioavailability, down-regulated miR-21 expression in chemo-resistant CRC cell lines by restoring PTEN levels and reducing Akt phosphorylation (Roy et al., 2013). Lastly, miR-21 suppression in CRC cells induced differentiation and increased their susceptibility to conventional (5-FU/oxaliplatin) or non-conventional (CDF) therapeutic regimens as well as their combination (Yu et al., 2013). In addition to miR-21, curcumin and CDF rescued the expression of miR-34 family members, lost in CRC, partly through demethylation of the respective promoters (Roy et al., 2012). Curcumin-mediated chemosensitization to 5-FU also occurred by up-regulation of epithelial–mesenchymal transition (EMT)-suppressive miR, including miR-34, further highlighting its potential therapeutic usefulness as an adjunct in patients with chemoresistant advanced CRC (Toden et al., 2015b). Likewise, key molecular mechanisms were identified when curcumin or boswellic acid (AKBA) were administered individually or in combination. These compounds synergized to affect specific miR and target genes involved in cell cycle regulation in CRC cell lines, including up-modulation of miR-34 and down-regulation of miR-27 ultimately leading to apoptosis induction, cell-cycle arrest and suppression of proliferation (Toden et al., 2015a). In line with these findings, curcumin or its most active synthetic analog RL197, inhibited CRC cell growth by ROS induction and reduction of Sp transcription factors and their regulators (i.e., ZBTB10 and ZBTB4) through miR-27a, miR-20a, and miR-17 (Gandhy et al., 2012), similarly to RES (Del Follo-Martinez et al., 2013). This regulation has important implications because Sp transcription factors regulate genes involved in cell death and angiogenesis and are often overexpressed in tumors. Moreover, curcumin is known to modulate DNA methylation in CRC cells, potentially exerting its anti-cancer effect by affecting other epigenetic mechanisms (Link et al., 2013).

ω3 PUFA found in walnuts, fish-oil, soybeans, green leafy vegetables, and seed oils are among dietary factors known to have an impact on miR involved in various stages of carcinogenesis, with a documented protective role in cancer, including CRC (Garcia-Segura et al., 2013). Conversely, ω6 PUFA (linoleic acid and arachidonic acid) found in vegetable oils and red meat, favor CRC onset (Abel et al., 2014). The protective effect of ω3 PUFA [docosahexaenoic acid (DHA) and eicosapentaenoic acid (EPA)] rich diets against CRC relies on their ability to modify gene expression and signaling pathways (Hou et al., 2016). In gastric cancer models, DHA and EPA have been reported to modulate apoptotic pathways. In fact ω3 PUFA treatment increased miR-15b and miR-16 and decreased miR-21, resulting in Bcl-2 down-regulation (Sun et al., 2013) and TNF-α up-regulation (Fluckiger et al., 2016), respectively. DHA also modulated the expression of specific miR (e.g., miR-30c and miR-192) in enterocytes, targeting genes related to lipid metabolism and cancer biology (Gil-Zamorano et al., 2014). In preclinical models, administration of fish oil- or walnut-enriched diets at early stages of carcinogenesis, modulated carcinogen-directed miR expression, as well as that of miR associated with inflammation, proliferation and apoptosis (Davidson et al., 2009; Tsoukas et al., 2015). Furthermore, combination of dietary fish oil and fermentable fiber pectin led to up-regulation of several miR, including miR-19b, miR-26b, and miR-203, whose validated targets (PDE4B, PTK2B, TCF4, IGF1R, and BACE1) promote tumorigenesis, as compared to control corn oil diet. Surprisingly, miR-21 was increased by the combination diet as compared to the control diet (Shah et al., 2011, 2016).

SCFA, such as acetate, butyrate, and propionate, represent additional protective metabolites produced by gut microbiota following fermentation of dietary fibers. Butyrate, a putative chemoprotective agent, acts as a histone deacetylase inhibitor (HDI) capable of decreasing proliferation and increasing apoptosis in CRC cells (Bultman, 2016). Studies have demonstrated that these effects are mediated in part through induction of CDKN1A expression (Crim et al., 2008) and by modulation of miR implicated in intestinal homeostasis and malignant transformation. Humphreys et al. (2013) explored the effects of butyrate and several other HDI on miR expression in human CRC cell lines. They reported that these HDI decrease miR-17∼92 cluster, while their target genes (e.g., PTEN, BCL2L11, CDKN1A) increase. Furthermore, butyrate induced expression of CDKN1A by suppressing members of the miR-106b family (Hu et al., 2011). Likewise, butyrate reduced the levels of pri-miR17-92a, precursor and mature miR-92a, as well as c-Myc, a main inducer of miR-17-92a promoter activity. This led to enhanced expression of CDKN1C (p57^KIP2^; Hu et al., 2015), one of the cyclin-dependent kinase inhibitors found dysregulated in cancer (Kavanagh and Joseph, 2011). As mentioned above, SCFA and fish oil ω3 PUFA worked coordinately *in vivo* to protect against colon tumorigenesis by modulating miR (Davidson et al., 2009; Shah et al., 2011, 2016). Collectively, these findings uncovered a novel mechanism whereby butyrate suppresses onco-miR biogenesis, promotes apoptosis and diminishes CRC cell proliferation.

## Promoting Effects

Although most bioactive nutrients exert a chemoprotective role in CRC, tumor-promoting effects have also been reported. Countries with high rates of overweight/obesity and consumption of red and processed meat and high fat intake show the highest CRC incidence (Bernstein et al., 2015). Conversely, CR is inversely associated with CRC risk and progression. The molecular mechanisms underlying these effects are being elucidated and include miR regulation of gene expression.

The effects of HFD and CR on miR expression were compared in a mouse CRC model (Olivo-Marston et al., 2014). Together with increased body weight and tumor numbers, HFD modulated miR expression in colonic mucosa, up-regulating onco-miR (e.g., miR-196 and miR-155) and concomitantly decreasing those involved in apoptosis regulation (e.g., miR-150). Interestingly, an opposite effect on tumor growth and miR expression was induced by CR diet (Olivo-Marston et al., 2014). Furthermore, in sporadic and inflammation-associated CRC models, HFD promoted weight gain and cancer development through EGFR-mediated induction of c-Myc and Kras (Dougherty et al., 2009). miR-143 and miR-145, negatively targeting these proto-oncogenes, were down-regulated by HFD in both tumor models only in EGFR-expressing mice, indicating that epigenetic changes associated with diet-induced colon tumorigenesis require EGFR signaling (Zhu et al., 2011). In a recent randomized dietary intervention study, the impact of high red meat (HRM) diet on rectal mucosa miR expression was examined (Humphreys et al., 2014). Short duration HRM consumption increased the levels of miR-17-92 cluster as well as miR-21, highly expressed in CRC and associated with poor survival. The enhanced miR-17-92 expression was associated with decreased levels of the target gene CDKN1A, and increased colonic cell proliferation. Supplementation with resistant starch, yielding high butyrate/propionate production when fermented, was able to reverse HRM effects on both miR-17-92 expression and cell proliferation. This study reported the first evidence in humans that HRM diet and resistant starch have opposite effects on rectal mucosa miR expression, supporting increased dietary fiber consumption as a mean for maintaining intestinal health and reducing HRM diet-associated CRC risk (Thompson, 2014). Likewise, dietary spinach had a protective effect when administered to rats together with heterocyclic amine 2-amino-1-methyl-6-phenylimidazo[4,5-b]pyridine (PhIP), a widely consumed carcinogen from cooked meat (Parasramka et al., 2012). This study demonstrated that a dietary carcinogen induces colon tumors with a signature loss of miR-215 and miR-let7 family members, whose targets (i.e., cyclin D1, HMGA2, β-catenin, c-Myc, p53) are known to promote EMT or regulate cell cycle. Once again, PhIP-induced down-modulation of miR expression, and concomitant increase in tumor incidence, were reversed after dietary spinach treatment, pointing to an important role of these latter in CRC chemoprevention.

Dietary components involved in one-carbon metabolism (folate, vitamin B12, cysteine, homocysteine) modulate miR expression and influence cancer risk by regulating DNA methylation pathways. Although a miR-mediated tumor protective role for folic acid has been reported in several cancers (Davis and Ross, 2008), in a recent study a pro-oncogenic effect was instead observed in human CRC (Beckett et al., 2015). Circulating folate levels directly correlated with serum miR-21 expression and with adenomatous polyps occurrence in females. Moreover, following stimulation of different CRC cell lines with excess folic acid, a significant increase in miR-21 release was found, suggesting a direct role for folate in driving tumor growth (Beckett et al., 2015). As serum miR-21 has been proposed as a CRC biomarker, these data suggest that dietary components/nutritional status may not only affect cancer development/progression via ncRNA modulation but also need to be considered when assessing the value of these molecules as possible biomarkers.

## Conclusion

**FIGURE 1:**
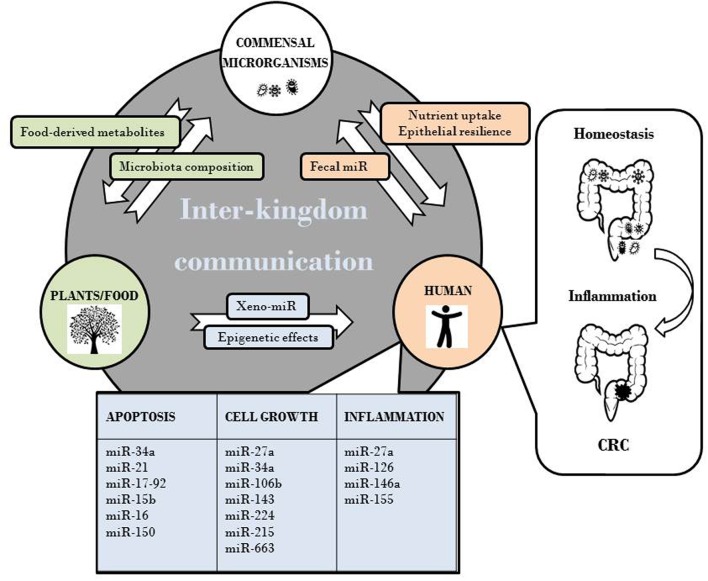
**Effects of plant-animal inter-kingdom communication on CRC development via miR deregulation**.

ncRNA are recognized epigenetic regulators with a well-established role in cancer (Di Leva et al., 2014). The potential cross-interaction among plant or microbial and human miR and their mRNA targets has been hypothesized by several authors and has been suggested to play a role in disease onset. The diet-mediated inter-kingdom signaling between plants and animals, object of this review, can be considered as part of a general vision of inter-kingdom communications mediated by ncRNA. As schematically depicted in **Figure 1**, plants and food, in addition to represent a source of xeno-miR, epigenetically influence host gene expression and potentially CRC development via miR deregulation. This supports the evidence that consumption of certain types of food is relevant for disease pathogenesis. Furthermore, diet indirectly affects the composition and metabolism of gut microbiota. In turn, microbiota influences nutrient uptake and epithelial resilience and drives the conversion of food components and fibers into metabolites that epigenetically regulate host gene expression. Of note, a cross-talk between human and gut microbiota through fecal miR has been recently highlighted (Celluzzi and Masotti, 2016). Overall we can envisage that each individual is placed in a complex inter-kingdom communication network that contributes to maintain homeostasis. Disruption of this equilibrium could set the basis for pathological states, for instance intestinal dysbiosis, inflammation and CRC development. Understanding the effects of dietary- and microbial-derived factors on ncRNA regulation will likely represent an important undertaken in human disease management. If successful, it may provide insights for the developing novel prevention strategies to reduce CRC burden.

## Author Contributions

MD, GD, LC, and SG contributed to the conception, writing, and editing of this manuscript. All authors read and approved the final manuscript.

## Conflict of Interest Statement

The authors declare that the research was conducted in the absence of any commercial or financial relationships that could be construed as a potential conflict of interest.
